# Efficient C‐to‐G editing in rice using an optimized base editor

**DOI:** 10.1111/pbi.13841

**Published:** 2022-06-03

**Authors:** Yifu Tian, Rundong Shen, Zuren Li, Qi Yao, Xuening Zhang, Dating Zhong, Xinhang Tan, Minglei Song, Han Han, Jian‐Kang Zhu, Yuming Lu

**Affiliations:** ^1^ Shanghai Center for Plant Stress Biology Center for Excellence in Molecular Plant Sciences Chinese Academy of Sciences Shanghai China; ^2^ 441102 Hunan Academy of Agricultural Sciences Changsha China; ^3^ School of Agriculture and Biology Shanghai Jiao Tong University Shanghai China; ^4^ Institute of Advanced Biotechnology, and School of Life Sciences Southern University of Science and Technology Shenzhen China; ^5^ Center for Advanced Bioindustry Technologies, and Institute of Crop Sciences Chinese Academy of Agricultural Sciences Beijing China; ^6^ Hainan Yazhou Bay Seed Lab Sanya Hainan China

**Keywords:** Cas9, base editing, rice, CGBE, UNG

Cytosine and adenosine base editors (CBE and ABE) have been vigorously developed in plants, but the base conversion types are limited (Ren *et al*., 2018, 2021; Xu *et al*., 2021). A new base editor CGBE with a uracil DNA N‐glycosylase (UNG) has recently been reported that enables efficient C‐to‐G editing in mammalian cells (Kurt *et al*., 2021). In plants, generating more base substitution types can expand its application, and help create new germplasm resources (Bharat *et al*., 2020; Butt *et al*., 2020; Molla and Yang, 2019). Currently, CGBEs applicable to plants have yet to be developed. Here, we established a CGBE system in rice that enables efficient C‐to‐G editing.

We firstly developed a reporter system to evaluate C‐to‐G/A editing. As shown in Figure 1a, the nano luciferase (nLuc) reporter was inactivated by a stop‐codon mutation. CBE induced C‐to‐T (TAG to TAA) cannot restore its function, but C‐to‐G/A edit would restore it to tyrosine (TAT/TAC), making it possible to evaluate the C‐to‐G/A activity. Accordingly, three sgRNAs were designed and the CBE editor PCBE4 was initially used for evaluation. Protoplasts‐based assays showed that this conventional CBE could mediate C‐to‐G/A editing, but at low efficiencies (0.11–0.14%, corresponding to 100% for the positive reporter, Figure 1b,c). This result illustrated the demand for efficiency improvement of C‐to‐G, and also validated the feasibility of this reporter system.

**Figure 1:**
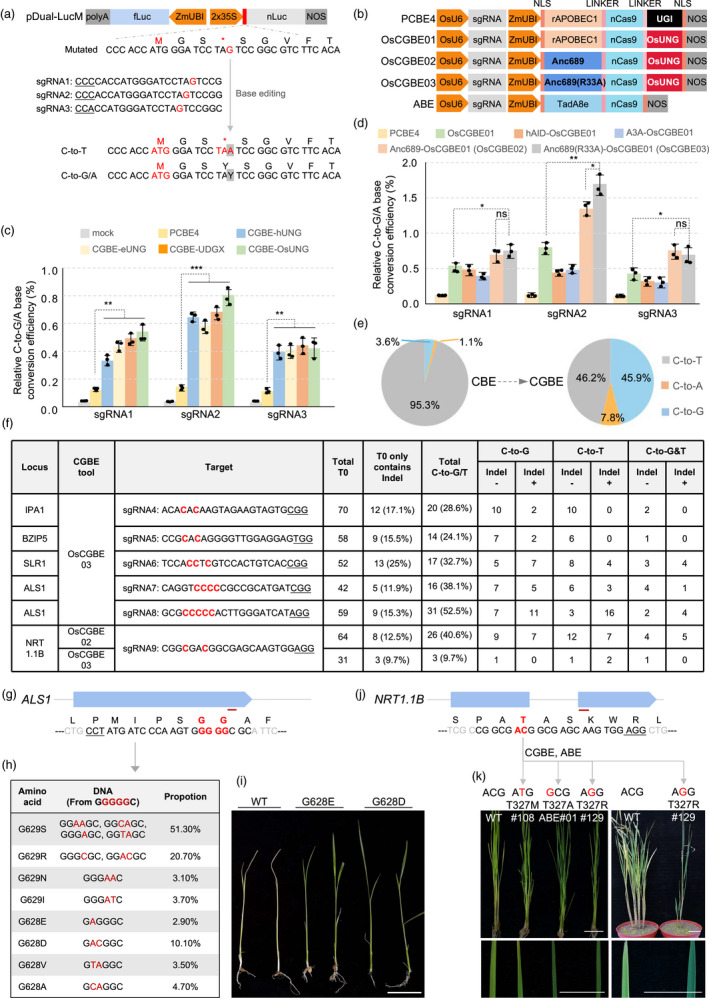
Development of CGBE editors in rice. (a) Dual‐luciferase reporter system for assessments of C‐to‐G/A editing. Nano luciferase (nluc) was inactivated by a stop‐codon mutation. fLuc, firefly luciferase. (b) Schematics of base editors used in this study. (c, d) Comparison of C‐to‐G/A conversion frequencies using CBE and CGBE editors fused with different UNGs (c) or cytosine deaminases (d). Rice protoplasts were co‐transformed with indicated base editor and pDual‐LucM. For all plots, values (mean ± SD) were calculated from three independent experiments (*n* = 3). *P* values were obtained using the two‐tailed Student’s *t*‐test. *P < 0.1, **P < 0.01. (e) Base conversion fractions of all targets at position C6. (f) Genotyping results of T0 plants edited using CGBE. Indel+/‐ indicated the number of plants with or without indel, respectively. (g, j) Diagram of *ALS1* and *NRT1.1B* with their sgRNAs. (h) Genotype results of *ALS1* T0 plants using Hi‐TOM, grouped by amino acid variations. (i) Phenotypes of T1 seedlings of *ALS1* after imazethapyr treatment. (k) Phenotypes of T0 plants edited with ABE (ABE#01) or CGBE (#108 and #129) at *NRT1.1B*. Seedlings (left) and 3‐month‐old plants (right) were grown in the greenhouse and enlarged views of their leaves are shown below. (i, k) Scale bar, 5 cm. WT, wild type. (a, f, g, j) PAMs are underlined and edited bases are marked in red.

We then synthesized three codon‐optimized UNGs from human (hUNG), E. *coli* (eUNG) and *Mycobacterium smegmatis* (UDGX), and constructed them into PCBE4 to replace the UGI and generated three CGBE vectors (Table S1). Protoplasts‐based assays revealed a substantial increase in the C‐to‐G/A editing efficiency compared with CBE (0.49% vs 0.12%), indicating C‐to‐G/A editing feasibility (Figure 1c). Since UNG is a conserved class of proteins, by sequence comparison, we identified LOC_Os04g57730 as a candidate (OsUNG) to generate another CGBE, CGBE‐OsUNG (Figure S1). Luciferase‐based assay showed that its C‐to‐G/A editing efficiency was further improved. We named CGBE‐OsUNG as OsCGBE01 for subsequent optimization.

Since CGBE may also depend on the efficiency of cytosine deamination, three highly active deaminases, hAID, hA3A, and Anc689, were selected to replace rAPOBEC1 in OsCGBE01. Luciferase assays showed that hAID or hA3A did not increase the efficiency, but Anc689 (named OsCGBE02, Addgene#183807) significantly improved the efficiency, with a 9.6‐fold enhancement at C6. We then tested another optimization on Anc689 with R33A, which resulted in a further improvement (OsCGBE03, Addgene#183808), reaching up to 1.69% at C6, 12.1 times higher than the original PCBE4 (Figure 1d).

Next, we tested OsCGBE03 on four rice genes (*OsIPA1*, *OsbZIP5, OsSLR1,* and *OsALS1*) to obtain stable edited plants (Figure 1f). A total of 128 T0 plants were firstly generated for *OsIPA1* and *OsbZIP5*. DSDecode analysis on Sanger sequencing results (Liu *et al*., 2015) showed that 21 and 16 plants contained C‐to‐G and C‐to‐T editing, respectively. We later used Hi‐TOM (Liu *et al*., 2019) to examine the 94 T0 plants for *SLR1* and *ALS1*. The results showed that more than half (65%) contained either C‐to‐G and/or C‐to‐T conversion (reads proportion >1% was counted, referred to as chimerism rate). To ensure heritability, we included only those plants with a chimerism rate >10% as valid edited plants. Accordingly, 24 plants (25.5%) were identified to harbour C‐to‐G conversions and 21 plants (22.3%) harbour C‐to‐T conversions. Biallelic plants with both conversions (C‐to‐T&G) were also frequently detected. Collectively, we achieved both C‐to‐G and C‐to‐T editing in a total of 222 plants at a high frequency (30.2%). When all base conversion types at C6 were analysed, we found that the proportion of C‐to‐G was dramatically increased from 3.6% to 45.9% (Figure 1e). These results demonstrated the feasibility of CGBE.

To explore its value in creating genetic diversity, we further edited *ALS1* and *NRT1.1B*. *ALS1* is responsible for herbicide resistant and sgRNA8 was designed targeting G628 (Figure 1g). Hi‐Tom sequencing for the 59 OsCGBE03‐edited plants showed that 31 lines (52.5%) contained C‐to‐G/T, resulting in a total of 8 types of amino acid substitutions (Figure 1h). Imazethapyr treatment (0.03%) showed that the G628E and G628D lines exhibited herbicide‐resistance phenotypes (Figure 1i). OsCGBE02, OsCGBE03, and an ABE with sgRNA‐9 were applied to edit *NRT1.1B* (Figure 1j). As expected, OsCGBE02 gave a higher C‐to‐G/T conversion frequency than OsCGBE03 at C7 (40.6% vs 9.7%), while both produced a more diverse editing pattern than ABE. Compared with the wild type, the novel variations (T327R or T327A) gave an obvious dark‐green phenotype similar to that of the natural‐occurring T327M allele, possibly reflecting the altered nitrogen use efficiency (Figure 1k). These case studies further demonstrated the value of CGBE in creating novel base‐substitutions for plant breeding.

Taken together, our CGBE editing on five rice genes produced a total of 376 T0 plants and C‐to‐G conversions were successful at all loci tested, with an average frequency of 21.3%. Genetic analysis of 11 lines confirmed a heritability ranging from 4.2% to 62.5% (Table S2). Our CGBE showed greater stability and higher editing efficiency in rice than the recently reported systems (Sretenovic *et al*., 2021). Compared to that in mammalian cells, it gave higher indel frequencies (111 lines, 29.5%, Figure S2), indicating that our CGBE needs to be further optimized in the future by inhibiting the base excision repair process.

## Author contributions

Y.L. and J.‐K.Z. designed the study. Y.T., R.S., and Z.L. performed most experiments. Z.L., Q.Y., and D.Z performed rice transformation. X.T., X.Z., and M.S genotyped the transgenic lines. Y.T., H.H., Y.L., and J.‐K.Z. wrote the manuscript.

## Conflict of interests

The authors declare no competing interests.

## Supporting information


**Figure S1** Sequence alignment for eUNG, 28 hUNG, UDGX and OsUNG.
**Figure S2** OsCGBE03 induced small fragment deletions in transgenic plants.
**Table S1** DNA sequences of related 39 vectors and genes.
**Table S2** Heritability analysis 42 on T1 progenies.
**Table S3** Oligos 44 used in this study.
