# Supplementary material for: Efficient C‐to‐G editing in rice using an optimized base editor
Source: Plant Biotechnol J. 2022 Jun 3;20(7):1238–40. doi: 10.1111/pbi.13841 (PMC9241366; doi:10.1111/pbi.13841)
Supplement: Supplementary file 1 — Figure S1 Sequence alignment for eUNG, 28 hUNG, UDGX and OsUNG. Figure S2 OsCGBE03 induced small fragment deletions in transgenic plants. Table S1 DNA sequences of related 39 vectors and genes. Table S2 Heritability analysis 42 on T1 progenies. Table S3 Oligos 44 used in this study. [file PBI-20-1238-s001.pdf]

Supplementary figures and tables for

Efficient C-to-G editing in rice using an optimized base editor

Yifu Tian[#,1], Rundong Shen[#,1], Zuren Li[#,2], Qi Yao[1], Xuening Zhang[1], Dating Zhong[1], Xinhang Tan[1], Minglei Song[1], Han Han[3], Jian-Kang Zhu[*,1,4,5,6] and Yuming Lu[*,1,3]

[1]Shanghai Center for Plant Stress Biology, Center for Excellence in Molecular Plant Sciences, Chinese Academy of Sciences, Shanghai 201602, China.

[2]Hunan Academy of Agricultural Sciences, Changsha 410125, China.

[3]School of Agriculture and Biology, Shanghai Jiao Tong University, Shanghai 200240, China.

[4]Institute of Advanced Biotechnology, and School of Life Sciences, Southern University of Science and Technology, Shenzhen 518055, China

[5]Center for Advanced Bioindustry Technologies, and Institute of Crop Sciences, Chinese Academy of Agricultural Sciences, Beijing 100081, China

[6]Hainan Yazhou Bay Seed Lab, Sanya, Hainan 572024, China

[#] These authors contribute equally.

[*] To whom correspondence should be addressed. E-mail: luymin@sjtu.edu.cn, zhujk@sustech.edu.cn.

Keywords: Cas9, base editing, rice, CGBE, UNG

Running title: C-to-G editing in rice

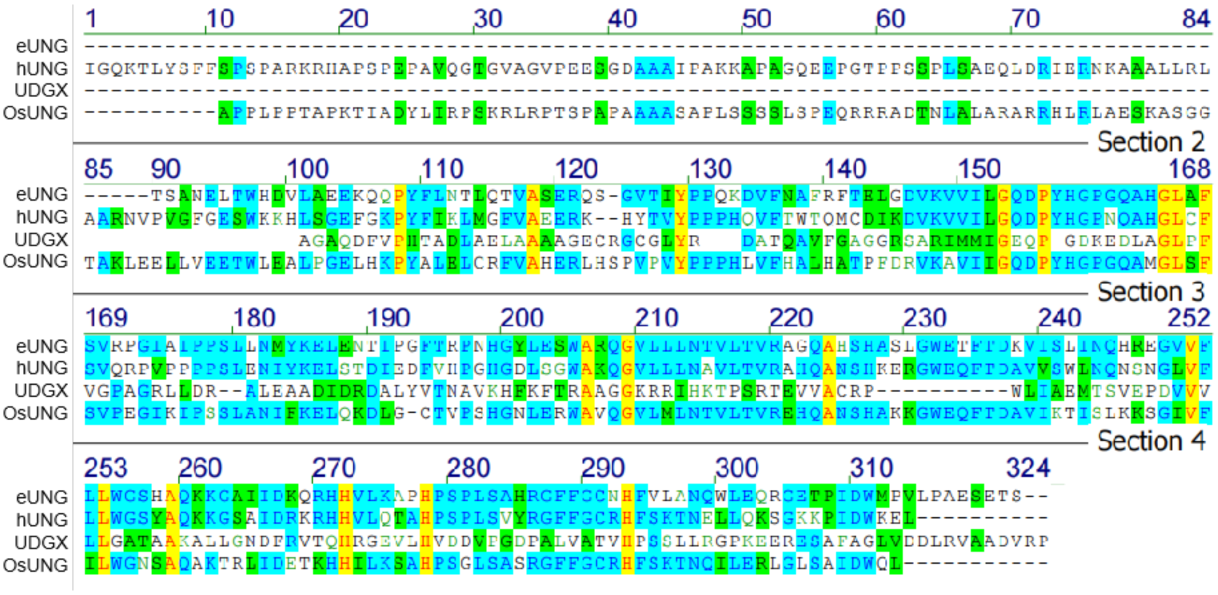

Figure S1 Sequence alignment for eUNG, hUNG, UDGX and OsUNG.

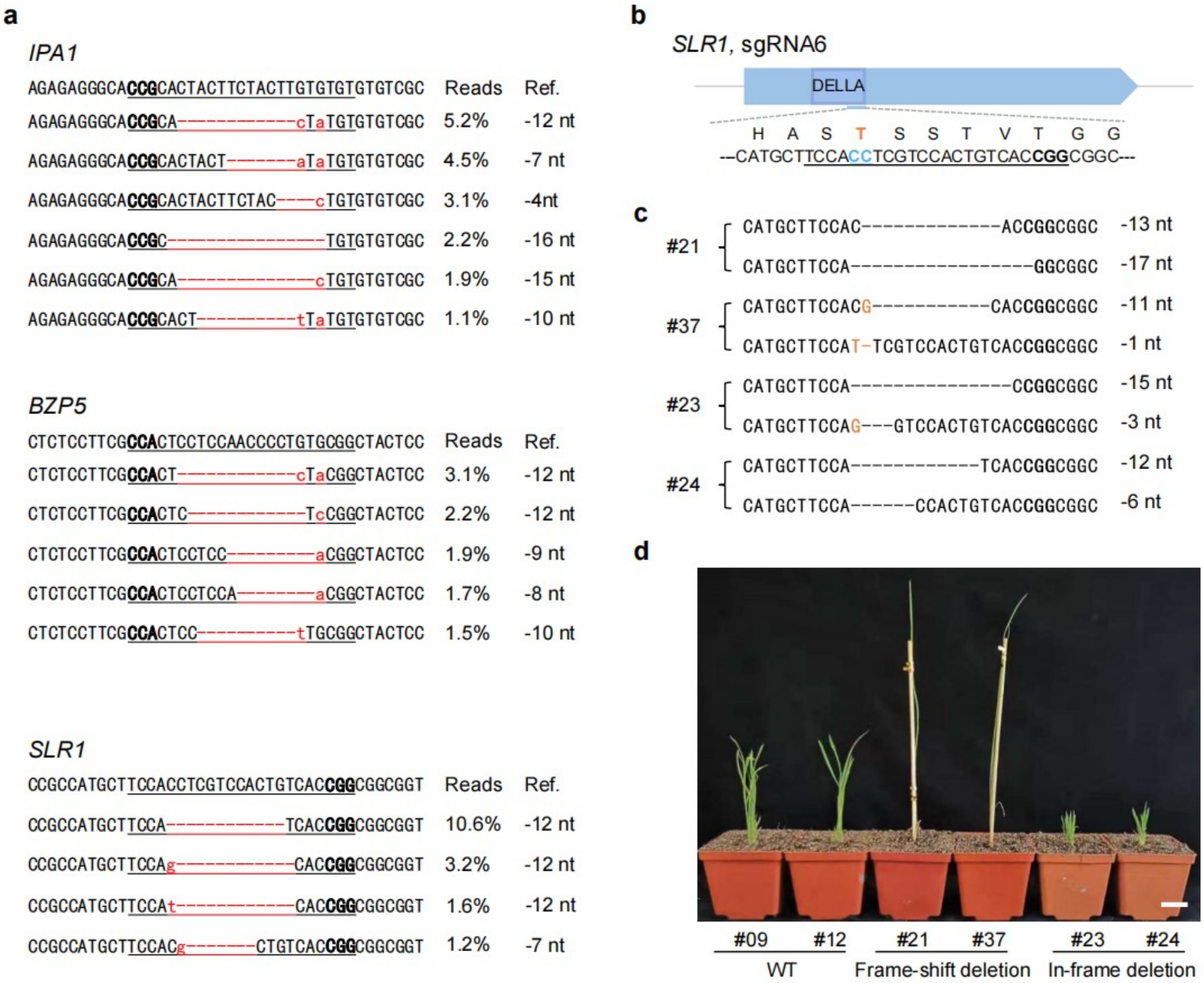

Figure S2 OsCGBE03 induced small fragment deletions in transgenic plants. a, Sequencing result of the CGBE-induced small fragment deletions in rice calli. **b,** Schematic to show the target site within DELLA domain of rice *SLR1*. **c,** Sequencing result of the CGBE-induced small fragment deletions in T0 transgenic seedlings. **d,** Phenotype of the regenerated T0 plants with frame-shift (#17, #45) and in-frame (#33, #34) deletions in the DELLA domain of *SLR1*. Scale bar, 3 cm. The spacer was underlined and the PAM motif was marked in bold. The targeted bases for deamination were marked in orange.

Table S1 DNA sequences of related vectors and genes

>pDual-LucM, 35S-Target-nLuc-NOS. Targeted base is marked in bold

AGCTTGCATGCCTGCAGGTCCCCAGATTAGCCTTTTCAATTTCAGAAAGAATGCTAACCCACAGATGGTTAGAGAGGCT
TACGCAGCAGGTCTCATCAAGACGATCTACCCGAGCAATAATCTCCAGGAAATCAAATACCTTCCCAAGAAGGTTAAAG
ATGCAGTCAAAAGATTCAGGACTAACTGCATCAAGAACACAGAGAAAGATATATTTCTCAAGATCAGAAGTACTATTCCA
GTATGGACGATTCAAGGCTTGCTTCACAAACCAAGGCAAGTAATAGAGATTGGAGTCTCTAAAAAGGTAGTTCCCACTG
AATCAAAGGCCATGGAGTCAAAGATTCAAATAGAGGACCTAACAGAACTCGCCGTAAAGACTGGCGAACAGTTCATACA
GAGTCTCTTACGACTCAATGACAAGAAGAAAATCTTCGTCAACATGGTGGAGCACGACACACTTGTCTACTCCAAAAAT
ATCAAAGATACAGTCTCAGAAGACCAAAGGGCAATTGAGACTTTTCAACAAAGGGTAATATCCGGAAACCTCCTCGGAT
TCCATTGCCCAGCTATCTGTCACTTTATTGTGAAGATAGTGGAAAAGGAAGGTGGCTCCTACAAATGCCATCATTGCGAT
AAAGGAAAGGCCATCGTTGAAGATGCCTCTGCCGACAGTGGTCCCAAAGATGGACCCCCACCCACGAGGAGCATCGT
GGAAAAAGAAGACGTTCCAACCACGTCTTCAAAGCAAGTGGATTGATGTGATATCTCCACTGACGTAAGGATGACGC
ACAATCCCACTATCCTTCGCAAGACCCTTCCTCTATATAAGGAAGTTCATTTCATTTGGAGAGAACACGGGGGACAAGC
TTGGTACCTCTAGACCCCACCATGGGATCCTA**G**TCCGGCGTCTTCACACTCGAAGATTTCGTTGGGGACTGGCGACAG
ACAGCCGGCTACAACCTGGACCAAGTCCTTGAACAGGGAGGTGTGTCCAGTTTGTTTCAGAATCTCGGGGTGTCCGT
AACTCCGATCCAAAGGATTGTCCTGAGCGGTGAAAATGGGCTGAAGATCGACATCCATGTCATCATCCCGTATGAAGG
TCTGAGCGGCGACCAAATGGGCCAGATCGAAAAAATTTTTAAGGTGGTGTACCCTGTGGATGATCATCACTTTAAGGTG
ATCCTGCACTATGGCACACTGGTAATCGACGGGGTTACGCCGAACATGATCGACTATTTCGGACGGCCGTATGAAGGC
ATCGCCGTGTTCGACGGCAAAAAGATCACTGTAACAGGGACCCTGTGGAACGGCAACAAAATTATCGACGAGCGCCT
GATCAACCCCGACGGCTCCCTGCTGTTCCGAGTAACCATCAACGGAGTGACCGGCTGGCGGCTGTGCGAACGCATT
CTGGCGTAAGGATCCCTCGAGCTCCTGCAGATCGTTCAAACATTTGGCAATAAAGTTTCTTAAGATTGAATCCTGTTGC
CGGTCTTGCGATGATTATCATATAATTTCTGTTGAATTACGTTAAGCATGTAATAATTAACATGTAATGCATGACGTTATTTA
TGAGATGGGTTTTTATGATTAGAGTCCCGCAATTATACATTTAATACGCGATAGAAAACAAAATATAGCGCGCAAACTAGG
ATAAATTATCGCGCGCGGTGTCATCTATGTTACTAGATCGG

>OsCGBE03, NLS- Anc689(**R33A**)-nCas9-OsUNG-NLS

ATGAAACGGACAGCCGACGGAAGCGAGTTCGAGTCACCAAAGAAGAAGCGGAAAGTCAGCAGTGAAACCGGACCAG
TGGCAGTGGACCCAACCCTGAGGAGACGGATTGAGCCCCATGAATTTGAAGTGTTCTTTGACCCAAGGGAGCTG**GCG**
AAGGAGACATGCCTGCTGTACGAGATCAAGTGGGGCACAAGCCACAAGATCTGGCGCCACAGCTCCAAGAACACCAC
AAAGCACGTGGAAGTGAATTTCATCGAGAAGTTTACCTCCGAGCGGCACTTCTGCCCCTCTACCAGCTGTTCCATCAC
ATGGTTTCTGTCTTGGAGCCCTTGCGGCGAGTGTTCCAAGGCCATCACCGAGTTCCTGTCTCAGCACCCTAACGTGA
CCCTGGTCATCTACGTGGCCCGGCTGTATCACCACATGGACCAGCAGAACAGGCAGGGCCTGCGCGATCTGGTGAAT
TCTGGCGTGACCATCCAGATCATGACAGCCCCAGAGTACGACTATTGCTGGCGGAACTTCGTGAATTATCCACCTGGC
AAGGAGGCACACTGGCCAAGATACCCACCCCTGTGGATGAAGCTGTATGCACTGGAGCTGCACGCAGGAATCCTGGG
CCTGCCTCCATGTCTGAATATCCTGCGGAGAAAGCAGCCCCAGCTGACATTTTTCACCATTGCTCTGCAGTCTTGTCAC
TATCAGCGGCTGCCTCCTCATATTCTGTGGGCTACAGGCCTTAAATCTGGAGGATCTAGCGGAGGCTCCTCTGGCAGC
GAGACACCAGGAACAAGCGAGTCAGCAACACCAGAGAGCAGTGGCGGCAGCAGCGGCGGATCCGACAAGAAGTAC
AGCATCGGCCTGGCCATCGGCACCAACTCTGTGGGCTGGGCCGTGATCACCGACGAGTACAAGGTGCCCAGCAAGA
AATTCAAGGTGCTGGGCAACACCGACCGGCACAGCATCAAGAAGAACCTGATCGGAGCCCTGCTGTTCGACAGCGG
CGAAACAGCCGAGGCCACCCGGCTGAAGAGAACCGCCAGAAGAAGATACACCAGACGGAAGAACCGGATCTGCTAT
CTGCAAGAGATCTTCAGCAACGAGATGGCCAAGGTGGACGACAGCTTCTTCCACAGACTGGAAGAGTCCTTCCTGGT
GGAAGAGGATAAGAAGCACGAGCGGCACCCCATCTTCGGCAACATCGTGGACGAGGTGGCCTACCACGAGAAGTAC

CCCACCATCTACCACCTGAGAAAGAAACTGGTGGACAGCACCGACAAGGCCGACCTGCGGCTGATCTATCTGGCCCT
GGCCCACATGATCAAGTTCCGGGGCCACTTCCTGATCGAGGGCGACCTGAACCCCGACAACAGCGACGTGGACAAG
CTGTTCATCCAGCTGGTGCAGACCTACAACCAGCTGTTCGAGGAAAACCCCATCAACGCCAGCGGCGTGGACGCCAA
GGCCATCCTGTCTGCCAGACTGAGCAAGAGCAGACGGCTGGAAAATCTGATCGCCCAGCTGCCCGGCGAGAAGAAG
AATGGCCTGTTCGGAAACCTGATTGCCCTGAGCCTGGGCCTGACCCCCAACTTCAAGAGCAACTTCGACCTGGCCGA
GGATGCCAAACTGCAGCTGAGCAAGGACACCTACGACGACGACCTGGACAACCTGCTGGCCCAGATCGGCGACCAG
TACGCCGACCTGTTTCTGGCCGCCAAGAACCTGTCCGACGCCATCCTGCTGAGCGACATCCTGAGAGTGAACACCGA
GATCACCAAGGCCCCCCTGAGCGCCTCTATGATCAAGAGATACGACGAGCACCACCAGGACCTGACCCTGCTGAAAG
CTCTCGTGCGGCAGCAGCTGCCTGAGAAGTACAAAGAGATTTTCTTCGACCAGAGCAAGAACGGCTACGCCGGCTAC
ATTGACGGCGGAGCCAGCCAGGAAGAGTTCTACAAGTTCATCAAGCCCATCCTGGAAAAGATGGACGGCACCGAGGA
ACTGCTCGTGAAGCTGAACAGAGAGGACCTGCTGCGGAAGCAGCGGACCTTCGACAACGGCAGCATCCCCCACCAG
ATCCACCTGGGAGAGCTGCACGCCATTCTGCGGCGGCAGGAAGATTTTTACCCATTCCTGAAGGACAACCGGGAAAA
GATCGAGAAGATCCTGACCTTCCGCATCCCCTACTACGTGGGCCCTCTGGCCAGGGGAAACAGCAGATTCGCCTGGA
TGACCAGAAAGAGCGAGGAAACCATCACCCCCTGGAACTTCGAGGAAGTGGTGGACAAGGGCGCTTCCGCCCAGAG
CTTCATCGAGCGGATGACCAACTTCGATAAGAACCTGCCCAACGAGAAGGTGCTGCCCAAGCACAGCCTGCTGTACG
AGTACTTCACCGTGTATAACGAGCTGACCAAAGTGAAATACGTGACCGAGGGAATGAGAAAGCCCGCCTTCCTGAGCG
GCGAGCAGAAAAAGGCCATCGTGGACCTGCTGTTCAAGACCAACCGGAAAGTGACCGTGAAGCAGCTGAAAGAGGA
CTACTTCAAGAAAATCGAGTGCTTCGACTCCGTGGAAATCTCCGGCGTGGAAGATCGGTTCAACGCCTCCCTGGGCA
CATACCACGATCTGCTGAAAATTATCAAGGACAAGGACTTCCTGGACAATGAGGAAAACGAGGACATTCTGGAAGATAT
CGTGCTGACCCTGACACTGTTTGAGGACAGAGAGATGATCGAGGAACGGCTGAAAACCTATGCCCACCTGTTCGACG
ACAAAGTGATGAAGCAGCTGAAGCGGCGGAGATACACCGGCTGGGGCAGGCTGAGCCGGAAGCTGATCAACGGCAT
CCGGGACAAGCAGTCCGGCAAGACAATCCTGGATTTCCTGAAGTCCGACGGCTTCGCCAACAGAAACTTCATGCAGC
TGATCCACGACGACAGCCTGACCTTTAAAGAGGACATCCAGAAAGCCCAGGTGTCCGGCCAGGGCGATAGCCTGCAC
GAGCACATTGCCAATCTGGCCGGCAGCCCCGCCATTAAGAAGGGCATCCTGCAGACAGTGAAGGTGGTGGACGAGC
TCGTGAAAGTGATGGGCCGGCACAAGCCCGAGAACATCGTGATCGAAATGGCCAGAGAGAACCAGACCACCCAGAA
GGGACAGAAGAACAGCCGCGAGAGAATGAAGCGGATCGAAGAGGGCATCAAAGAGCTGGGCAGCCAGATCCTGAAA
GAACACCCCGTGGAAAACACCCAGCTGCAGAACGAGAAGCTGTACCTGTACTACCTGCAGAATGGGCGGGATATGTA
CGTGGACCAGGAACTGGACATCAACCGGCTGTCCGACTACGATGTGGACCATATCGTGCCTCAGAGCTTTCTGAAGG
ACGACTCCATCGACAACAAGGTGCTGACCAGAAGCGACAAGAACCGGGGCAAGAGCGACAACGTGCCCTCCGAAGA
GGTCGTGAAGAAGATGAAGAACTACTGGCGGCAGCTGCTGAACGCCAAGCTGATTACCCAGAGAAAGTTCGACAATC
TGACCAAGGCCGAGAGAGGCGGCCTGAGCGAACTGGATAAGGCCGGCTTCATCAAGAGACAGCTGGTGGAAACCCG
GCAGATCACAAAGCACGTGGCACAGATCCTGGACTCCCGGATGAACACTAAGTACGACGAGAATGACAAGCTGATCC
GGGAAGTGAAAGTGATCACCCTGAAGTCCAAGCTGGTGTCCGATTTCCGGAAGGATTTCCAGTTTTACAAAGTGCGCG
AGATCAACAACTACCACCACGCCCACGACGCCTACCTGAACGCCGTCGTGGGAACCGCCCTGATCAAAAAGTACCCT
AAGCTGGAAAGCGAGTTCGTGTACGGCGACTACAAGGTGTACGACGTGCGGAAGATGATCGCCAAGAGCGAGCAGG
AAATCGGCAAGGCTACCGCCAAGTACTTCTTCTACAGCAACATCATGAACTTTTTCAAGACCGAGATTACCCTGGCCAA
CGGCGAGATCCGGAAGCGGCCTCTGATCGAGACAAACGGCGAAACCGGGGAGATCGTGTGGGATAAGGGCCGGGAT
TTTGCCACCGTGCGGAAAGTGCTGAGCATGCCCCAAGTGAATATCGTGAAAAAGACCGAGGTGCAGACAGGCGGCTT
CAGCAAAGAGTCTATCCTGCCCAAGAGGAACAGCGATAAGCTGATCGCCAGAAAGAAGGACTGGGACCCTAAGAAGT
ACGGCGGCTTCGACAGCCCCACCGTGGCCTATTCTGTGCTGGTGGTGGCCAAAGTGGAAAAGGGCAAGTCCAAGAA
ACTGAAGAGTGTGAAAGAGCTGCTGGGGATCACCATCATGGAAAGAAGCAGCTTCGAGAAGAATCCCATCGACTTTCT
GGAAGCCAAGGGCTACAAAGAAGTGAAAAAGGACCTGATCATCAAGCTGCCTAAGTACTCCCTGTTCGAGCTGGAAAA
CGGCCGGAAGAGAATGCTGGCCTCTGCCGGCGAACTGCAGAAGGGAAACGAACTGGCCCTGCCCTCCAAATATGTG
AACTTCCTGTACCTGGCCAGCCACTATGAGAAGCTGAAGGGCTCCCCCGAGGATAATGAGCAGAAACAGCTGTTTGTG

GAACAGCACAAGCACTACCTGGACGAGATCATCGAGCAGATCAGCGAGTTCTCCAAGAGAGTGATCCTGGCCGACGC
TAATCTGGACAAAGTGCTGTCCGCCTACAACAAGCACCGGGATAAGCCCATCAGAGAGCAGGCCGAGAATATCATCCA
CCTGTTTACCCTGACCAATCTGGGAGCCCCTGCCGCCTTCAAGTACTTTGACACCACCATCGACCGGAAGAGGTACAC
CAGCACCAAAGAGGTGCTGGACGCCACCCTGATCCACCAGAGCATCACCGGCCTGTACGAGACACGGATCGACCTG
TCTCAGCTGGGAGGCGACAGCGGCGGGAGCGGCGGGAGCGGCGGGAGCGGGGGGAGCGCGCCGCCTCTCCCTC
CCACCGCCCCCAAAACCATCGCCGACTACCTCATCCGCCCCTCCAAGCGCCTCCGCCCCACCTCTCCCGCTCCCGC
CGCCGCTGCGTCGGCCCCCCTCTCCTCCTCCAGCCTCTCGCCGGAGCAGCGCCGCCGCGCCGACACCAACCTCGC
GCTCGCCCGGGCGCGCCGCCACCTCCGCCTCGCCGAGTCCAAAGCGTCGGGCGGCACCGCGAAGCTGGAGGAGC
TGCTCGTCGAGGAGACATGGCTGGAGGCGCTTCCCGGGGAGCTGCACAAACCCTACGCGCTCGAACTCTGCCGCTT
CGTCGCCCACGAGAGGTTGCATAGCCCGGTGCCCGTCTACCCGCCGCCGCATCTAGTGTTCCACGCGCTTCACGCC
ACCCCGTTCGACCGTGTTAAGGCCGTCATCATCGGGCAGGACCCATACCACGGACCTGGTCAGGCGATGGGGTTGTC
TTTCTCAGTACCAGAGGGGATCAAAATTCCTTCTAGCTTAGCAAACATATTTAAAGAGCTGCAAAAAGATCTAGGTTGCA
CCGTGCCTTCACATGGAAACTTGGAAAGATGGGCTGTGCAGGGTGTTCTTATGCTCAACACTGTATTAACTGTGAGAG
AACATCAAGCCAATTCACATGCCAAGAAAGGATGGGAGCAATTTACTGATGCTGTCATTAAGACAATATCACTGAAGAAA
TCTGGAATAGTCTTTATTCTCTGGGGAAACTCAGCTCAAGCAAAGACAAGATTGATTGATGAAACAAAACACCACATTTT
GAAATCCGCTCATCCATCAGGGCTGTCTGCAAGCAGAGGTTTCTTTGGATGCAGGCACTTTTCTAAAACGAATCAGATC
TTGGAGAGGCTGGGACTATCTGCCATTGATTGGCAACTCTCTGGCGGCTCAAAAAGGCCGGCGGCCACGAAAAAGGC
CGGCCAGGCAAAAAAGAAAAAGTAG

>hUNG
ATCGGCCAGAAAACCCTGTACAGCTTCTTCAGCCCATCTCCTGCCAGAAAGCGGCACGCCCCATCTCCAGAACCTGC
TGTTCAAGGCACAGGCGTGGCAGGCGTGCCAGAAGAATCTGGCGACGCTGCTGCCATTCCTGCCAAAAAAGCCCCT
GCCGGCCAAGAGGAACCTGGCACACCTCCATCTTCTCCACTGTCTGCCGAGCAGCTGGACCGGATCGAGAGAAACA
AAGCCGCCGCTCTGCTGAGACTGGCCGCCAGAAATGTGCCTGTTGGCTTTGGCGAGAGCTGGAAGAAGCACCTGTC
TGGCGAGTTCGGCAAGCCCTACTTCATCAAGCTGATGGGCTTCGTGGCCGAGGAACGGAAGCACTACACAGTGTACC
CTCCACCTCACCAGGTGTTCACCTGGACACAGATGTGCGACATCAAGGACGTGAAGGTGGTCATCCTCGGACAGGAC
CCTTATCACGGCCCTAATCAGGCCCACGGCCTGTGCTTTAGCGTGCAAAGACCTGTGCCTCCTCCACCTAGCCTGGAA
AACATCTACAAAGAGCTGAGCACCGACATCGAGGACTTCGTGCATCCTGGACACGGCGATCTGTCTGGATGGGCTAAA
CAGGGCGTGCTGCTGCTGAATGCCGTGCTGACAGTTAGAGCCCACCAGGCCAACAGCCACAAAGAGAGAGGCTGGG
AGCAGTTCACCGATGCCGTGGTGTCTTGGCTGAACCAGAACAGCAACGGCCTGGTGTTTCTGCTGTGGGGCAGCTAC
GCCCAGAAGAAGGGAAGCGCCATCGACCGGAAGAGACACCATGTGCTGCAGACAGCTCATCCATCTCCACTGAGCG
TGTACCGGGGCTTCTTCGGCTGTAGACACTTCAGCAAGACCAACGAGCTGCTGCAGAAGTCCGGCAAGAAGCCTATC
GACTGGAAAGAGCTG

>eUNG
ACTAGTGCCAACGAACTGACCTGGCACGACGTGCTGGCCGAAGAGAAGCAGCAGCCCTATTTCCTTAACACCCTGCA
GACCGTGGCCAGCGAGCGGCAGAGCGGCGTGACCATCTACCCCCCACAGAAAGACGTGTTTAACGCCTTCCGCTTC
ACAGAGCTGGGCGACGTGAAGGTGGTGATCCTGGGCCAGGACCCCTACCACGGCCCCGGCCAGGCCCATGGTCTG
GCCTTCAGCGTGCGGCCCGGCATCGCCATCCCCCCCAGCCTGCTGAACATGTATAAGGAGCTGGAAAACACCATCCC
CGGCTTCACCCGGCCCAATCACGGCTACCTGGAGAGCTGGGCGCGGCAGGGCGTGCTGCTGCTCAACACCGTGCT
GACGGTACGGGCCGGCCAGGCGCATAGCCACGCCAGCCTGGGCTGGGAGACGTTCACCGATAAGGTGATCAGCCTG
ATCAACCAGCACCGGGAGGGCGTGGTGTTTCTGCTGTGGGGCAGCCATGCCCAGAAGAAAGGCGCCATCATCGACA
AGCAGCGGCATCATGTGCTGAAAGCCCCCCACCCCAGCCCCCTTAGCGCCCACCGGGGCTTCTTCGGCTGCAACCA

TTTCGTGCTGGCCAATCAGTGGCTGGAACAACGGGGCGAGACGCCCATTGACTGGATGCCCGTGTTGCCCGCCGAG
AGCGAGACTAGT

>UDGX
GCAGGAGCACAGGATTTCGTTCCACATACCGCTGACCTTGCTGAACTGGCCGCAGCTGCGGGTGAGTGTAGAGGATG
CGGCTTGTATCGCGATGCGACTCAGGCGGTATTTGGCGCTGGCGGTAGGTCTGCGAGAATCATGATGATCGGAGAAC
AGCCGGGTGACAAAGAGGACCTCGCAGGTCTGCCTTTCGTGGGACCAGCTGGAAGGCTGTTGGATCGCGCTTTGGA
GGCAGCAGACATCGACAGGGATGCCCTGTACGTCACTAACGCCGTCAAACACTTCAAGTTTACACGCGCAGCAGGCG
GAAAGAGACGGATTCATAAGACTCCCTCCCGCACCGAAGTTGTTGCCTGTAGACCTTGGCTCATCGCGGAAATGACCT
CTGTTGAGCCGGATGTCGTCGTGCTTCTTGGAGCAACTGCGGCCAAGGCCTTGCTTGGCAACGATTTCCGCGTCACT
CAACACAGGGGCGAAGTTCTCCACGTTGACGACGTGCCAGGTGATCCTGCGCTCGTAGCCACAGTTCATCCTTCTTC
ACTCTTGAGGGGCCCGAAAGAAGAACGTGAGTCCGCCTTCGCCGGTTTGGTGGACGATCTGAGAGTTGCGGCGGAT
GTGCGCCCC

>OsUNG
GCGCCGCCTCTCCCTCCCACCGCCCCCAAAACCATCGCCGACTACCTCATCCGCCCCTCCAAGCGCCTCCGCCCCA
CCTCTCCCGCTCCCGCCGCCGCTGCGTCGGCCCCCCTCTCCTCCTCCAGCCTCTCGCCGGAGCAGCGCCGCCGCG
CCGACACCAACCTCGCGCTCGCCCGGGCGCGCCGCCACCTCCGCCTCGCCGAGTCCAAAGCGTCGGGCGGCACC
GCGAAGCTGGAGGAGCTGCTCGTCGAGGAGACATGGCTGGAGGCGCTTCCCGGGGAGCTGCACAAACCCTACGCG
CTCGAACTCTGCCGCTTCGTCGCCCACGAGAGGTTGCATAGCCCGGTGCCCGTCTACCCGCCGCCGCATCTAGTGTT
CCACGCGCTTCACGCCACCCCGTTCGACCGTGTTAAGGCCGTCATCATCGGGCAGGACCCATACCACGGACCTGGTC
AGGCGATGGGGTTGTCTTTCTCAGTACCAGAGGGGATCAAAATTCCTTCTAGCTTAGCAAACATATTTAAAGAGCTGCA
AAAAGATCTAGGTTGCACCGTGCCTTCACATGGAAACTTGGAAAGATGGGCTGTGCAGGGTGTTCTTATGCTCAACAC
TGTATTAACTGTGAGAGAACATCAAGCCAATTCACATGCCAAGAAAGGATGGGAGCAATTTACTGATGCTGTCATTAAGA
CAATATCACTGAAGAAATCTGGAATAGTCTTTATTCTCTGGGGAAACTCAGCTCAAGCAAAGACAAGATTGATTGATGAA
ACAAAACACCACATTTTGAAATCCGCTCATCCATCAGGGCTGTCTGCAAGCAGAGGTTTCTTTGGATGCAGGCACTTTT
CTAAAACGAATCAGATCTTGGAGAGGCTGGGACTATCTGCCATTGATTGGCAACTC

>hAID
GACAGCCTCTTGATGAACCGGAGGGAGTTTCTTTACCAATTCAAAAATGTCCGCTGGGCTAAGGGTCGGCGTGAGAC
GTACCTGTGCTACGTAGTGAAGAGGCGTGACAGTGCTACATCCTTTTCACTGGACTTTGGTTATCTTCGCAATAAGAAC
GGCTGCCACGTGGAATTGCTCTTCCTCCGCTACATCTCGGACTGGGACCTAGACCCTGGCCGCTGCTACCGCGTCAC
CTGGTTCATCTCCTGGAGCCCCTGCTACGACTGTGCCCGACATGTGGCCGACTTTCTGCGAGGGAACCCCAACCTCA
GTCTGAGGATCTTCACCGCGCGCCTCTACTTCTGTGAGGACCGCAAGGCTGAGCCCGAGGGGCTGCGGCGGCTGCA
CCGCGCCGGGGTGCAAATAGCCATCATGACCTTCAAAGATTATTTTTACTGCTGGAATACTTTTGTAGAAAACCACGGA
AGAACTTTCAAAGCCTGGGAAGGGCTGCATGAAAATTCAGTTCGTCTCTCCAGACAGCTTCGGCGCATCCTTTTGCCC
CTGTATGAGGTTGATGACTTACGAGACGCATTTCGTACT

>hAPOBEC3A
GAGGCCAGCCCGGCTAGCGGCCCAAGGCATCTCATGGACCCGCACATCTTCACCAGCAACTTCAACAACGGCATCGG
CAGGCACAAGACCTACTTGTGCTACGAGGTGGAGAGGCTCGACAACGGAACCTCCGTGAAGATGGACCAACACAGG
GGGTTCCTCCACAACCAAGCCAAGAACCTCCTCTGCGGCTTCTACGGCAGGCACGCCGAGTTGAGGTTCCTCGACTT
GGTGCCATCCCTCCAACTCGATCCAGCCCAAATCTACCGCGTGACCTGGTTCATCTCCTGGTCCCCATGCTTCTCCTG

GGGTTGCGCCGGCGAGGTTCGGGCTTTCCTCCAAGAAAACACCCACGTCCGCCTCCGCATTTTCGCCGCCAGGATCT
ATGATTACGACCCTCTCTACAAGGAGGCCCTCCAGATGCTGCGGGACGCCGGTGCTCAGGTGAGTATCATGACCTAC
GACGAGTTCAAGCACTGCTGGGACACCTTCGTTGACCACCAGGGCTGCCCATTCCAACCGTGGGACGGTCTGGATG
AACACAGCCAAGCCTTGTCCGGCAGGCTCCGGGCCATCCTCCAAAACCAGGGGAAC

>Anc689
AGCAGTGAAACCGGACCAGTGGCAGTGGACCCAACCCTGAGGAGACGGATTGAGCCCCATGAATTTGAAGTGTTCTT
TGACCCAAGGGAGCTGAGGAAGGAGACATGCCTGCTGTACGAGATCAAGTGGGGCACAAGCCACAAGATCTGGCGC
CACAGCTCCAAGAACACCACAAAGCACGTGGAAGTGAATTTCATCGAGAAGTTTACCTCCGAGCGGCACTTCTGCCC
CTCTACCAGCTGTTCCATCACATGGTTTCTGTCTTGGAGCCCTTGCGGCGAGTGTTCCAAGGCCATCACCGAGTTCCT
GTCTCAGCACCCTAACGTGACCCTGGTCATCTACGTGGCCCGGCTGTATCACCACATGGACCAGCAGAACAGGCAGG
GCCTGCGCGATCTGGTGAATTCTGGCGTGACCATCCAGATCATGACAGCCCCAGAGTACGACTATTGCTGGCGGAACT
TCGTGAATTATCCACCTGGCAAGGAGGCACACTGGCCAAGATACCCACCCCTGTGGATGAAGCTGTATGCACTGGAGC
TGCACGCAGGAATCCTGGGCCTGCCTCCATGTCTGAATATCCTGCGGAGAAAGCAGCCCCAGCTGACATTTTTCACCA
TTGCTCTGCAGTCTTGTCACTATCAGCGGCTGCCTCCTCATATTCTGTGGGCTACAGGCCTTAAA

>Anc689(R33A)
AGCAGTGAAACCGGACCAGTGGCAGTGGACCCAACCCTGAGGAGACGGATTGAGCCCCATGAATTTGAAGTGTTCTT
TGACCCAAGGGAGCTGGCGAAGGAGACATGCCTGCTGTACGAGATCAAGTGGGGCACAAGCCACAAGATCTGGCGC
CACAGCTCCAAGAACACCACAAAGCACGTGGAAGTGAATTTCATCGAGAAGTTTACCTCCGAGCGGCACTTCTGCCC
CTCTACCAGCTGTTCCATCACATGGTTTCTGTCTTGGAGCCCTTGCGGCGAGTGTTCCAAGGCCATCACCGAGTTCCT
GTCTCAGCACCCTAACGTGACCCTGGTCATCTACGTGGCCCGGCTGTATCACCACATGGACCAGCAGAACAGGCAGG
GCCTGCGCGATCTGGTGAATTCTGGCGTGACCATCCAGATCATGACAGCCCCAGAGTACGACTATTGCTGGCGGAACT
TCGTGAATTATCCACCTGGCAAGGAGGCACACTGGCCAAGATACCCACCCCTGTGGATGAAGCTGTATGCACTGGAGC
TGCACGCAGGAATCCTGGGCCTGCCTCCATGTCTGAATATCCTGCGGAGAAAGCAGCCCCAGCTGACATTTTTCACCA
TTGCTCTGCAGTCTTGTCACTATCAGCGGCTGCCTCCTCATATTCTGTGGGCTACAGGCCTTAAA

Table S2 Heritability analysis on T1 progenies

Locus	Parent			Progeny		
	Name	Generation	Genotype	Total	Base editing	Transmission
SLR1	SLR1-SG6#03	T0	WT	24	0	0.00%
	SLR1-SG6#10	T0	Chimeric	24	8	33.33%
	SLR1-SG6#27	T0	Chimeric	24	14	58.33%
	SLR1-SG6#48	T0	Chimeric	24	1	4.17%
ALS1	ALS1-SG7#02	T0	WT	24	0	0.00%
	ALS1-SG7#07	T0	Chimeric	24	7	29.17%
	ALS1-SG7#10	T0	Chimeric	24	3	12.50%
	ALS1-SG7#28	T0	Chimeric	24	2	8.33%
NRT1.1B	NRT-SG9#102	T0	WT	24	1	4.17%
	NRT-SG9#103	T0	Chimeric	24	2	8.33%
	NRT-SG9#104	T0	Chimeric	24	12	50.00%
	NRT-SG9#125	T0	Chimeric	24	11	45.83%
	NRT-SG9#129	T0	Chimeric	24	15	62.50%
	NRT-SG9#133	T0	Chimeric	24	13	54.17%

Table S3 Oligos used in this study

Primer Name	Primer Sequence	Application
IPA1-HF	GGAGTGAGTACGGTGTGCTTTTTAAGGGCGGAGAGAGAGAGAGAGAGAGGGCACCGCAC	1st PCR for Hi-TOM sequencing of sgRNA-4 target site
IPA1-HR	GAGTTGGATGCTGGATGGAAGAGAGAGGAAAGAGACGGAAAGGTGGCGAAGCCCAGCGA	
BZP5-HF	GGAGTGAGTACGGTGTGCCGCGCGCGGGCGGCGGCGAGCTCCCTCTCCTTCGCCACTCC	1st PCR for Hi-TOM sequencing of sgRNA-5 target site
BZP5-HR	GAGTTGGATGCTGGATGGAGAGAGCCACTTAGAACATGCTCG	
SLR1-HF	GGAGTGAGTACGGTGTGCAGCATGCTTTCCGAGCTCAACGC	1st PCR for Hi-TOM sequencing of sgRNA-6 target site
SLR1-HR	GAGTTGGATGCTGGATGGGGCAGCGGCTGGGAGTTCA	
ALS1-HF	GGAGTGAGTACGGTGTGCGCGCTGCTCGACTCCGT	1st PCR for Hi-TOM sequencing of sgRNA-7 target site
ALS1-HR	GAGTTGGATGCTGGATGGAGCGGGTGACCTCGACTAT	
ALS2-HF	GGAGTGAGTACGGTGTGCGCCATACTTGTTGGATATCATCGTC	1st PCR for Hi-TOM sequencing of sgRNA-8 target site
ALS2-HR	GAGTTGGATGCTGGATGGTTAATACACAGTCCTGCCATCACC	
NRT-HF	GGAGTGAGTACGGTGTGCTGCAGGTTCCTGGACCATGCG	1st PCR for Hi-TOM sequencing of sgRNA-9 target site
NRT-HR	GAGTTGGATGCTGGATGGCTGCTTCACCTCCTCCACGTC	
IPA1-F1	GTGCAGTGTCATTTAGAGTTCCCAA	PCR for Sanger sequencing of sgRNA-4 target site
IPA1-R1	CCACGCCCTTCCCCTTGTTCT	
BZP5-F1	CCCGGTGGCCTAGTCCTTATC	PCR for Sanger sequencing of sgRNA-5 target site
BZP5-R1	ACGGACTAATAATTTGGACGGAGTGAA	
SLR1-F1	CGGGTACAAGGTGCGGTCGTC	PCR for Sanger sequencing of sgRNA-6 target site
SLR1-R1	CCAGTGCGCATCCGCTTGG	
ALS1-F1	AGCAGGGCGAGGCGTTC	PCR for Sanger sequencing of sgRNA-7 target site
ALS1-R1	CGCCAACCAGACGCAAGACCT	
ALS2-F1	CAATATTCCTGCAGTCCGTGTAACA	PCR for Sanger sequencing of sgRNA-8 target site
ALS2-R1	ACCTAGACAGCAGGAAGCTAATT	
NRT-F1	ACTTTGGCTGCCAAGAAACACCACA	PCR for Sanger sequencing of sgRNA-9 target site
NRT-R1	CGTAGAAGGGGACGGTGAGGAGGAT	
sgRNA1-UP	tgtgtgGGACTAGGATCCCATGGTG	Construction of CGBE plasmid
sgRNA1-LW	aaacCACCATGGGATCCTAGTCCca	
sgRNA2-UP	tgtgtgCGGACTAGGATCCCATGGT	
sgRNA2-LW	aaacACCATGGGATCCTAGTCCGca	
sgRNA3-UP	tgtgtgCCGGACTAGGATCCCATGG	
sgRNA3-LW	aaacCCATGGGATCCTAGTCCGGca	
sgRNA4-UP	tgtgtgCACACAAGTAGAAGTAGTG	

sgRNA4-LW	aaacCACTACTTCTACTTGTGTGca	
sgRNA5-UP	tgtgtgCGCACAGGGGTTGGAGGAG	
sgRNA5-LW	aaacCTCCTCCAACCCCTGTGCGca	
sgRNA6-UP	tgtgtgCCACCTCGTCCACTGTCAC	
sgRNA6-LW	aaacGTGACAGTGGACGAGGTGGca	
sgRNA7-UP	tgtgtgAGGTCCCCCGCCGCATGAT	
sgRNA7-LW	aaacATCATGCGGCGGGGGACCTca	
sgRNA8-UP	tgtgtgCGCCCCCACTTGGGATCAT	
sgRNA8-LW	aaacATGATCCCAAGTGGGGGCGca	
sgRNA9-UP	tgtgtgGGCGACGGCGAGCAAGTGG	
sgRNA9-LW	aaacCCACTTGCTCGCCGTCGCCca	
